# Allografts for Skin Closure during In Utero Spina Bifida Repair in a Sheep Model

**DOI:** 10.3390/jcm10214928

**Published:** 2021-10-25

**Authors:** Lovepreet K. Mann, Jong Hak Won, Rajan Patel, Eric P. Bergh, Jeannine Garnett, Meenakshi B. Bhattacharjee, Ponnada A. Narayana, Ranu Jain, Stephen A. Fletcher, Dejian Lai, Ramesha Papanna

**Affiliations:** 1 Division of Maternal-Fetal Medicine, The Fetal Center at Children’s Memorial Hermann Hospital, Department of Obstetrics, Gynecology and Reproductive Medicine, McGovern Medical School, The University of Texas Health Science Center at Houston (UTHealth), Houston, TX 77030, USA; jong.hak.won@uth.tmc.edu (J.H.W.); eric.p.bergh@uth.tmc.edu (E.P.B.); jeannine.garnett@uth.tmc.edu (J.G.); 2 Department of Diagnostic and Interventional Imaging, McGovern Medical School, The University of Texas Health Science Center at Houston (UTHealth), Houston, TX 77030, USA; rajan.p.patel@uth.tmc.edu (R.P.); ponnada.a.narayana@uth.tmc.edu (P.A.N.); 3 Department of Pathology and Laboratory Medicine, McGovern Medical School, The University of Texas Health Science Center at Houston (UTHealth), Houston, TX 77030, USA; meenakshi.b.bhattacharjee@uth.tmc.edu; 4 Department of Anesthesia, McGovern Medical School, The University of Texas Health Science Center at Houston (UTHealth), Houston, TX 77030, USA; ranu.jain@uth.tmc.edu; 5 Division of Pediatric Neurosurgery, Department of Pediatric Surgery, McGovern Medical School, The University of Texas Health Science Center at Houston (UTHealth), Houston, TX 77030, USA; stephen.fletcher@uth.tmc.edu; 6 Department of Biostatistics, School of Public Health, The University of Texas Health Science Center at Houston (UTHealth), Houston, TX 77030, USA; dejian.lai@uth.tmc.edu

**Keywords:** umbilical cord, regenerative healing, sheep spina bifida repair model, inflammation, astrocyte activation, acellular dermal matrix, conventional repair

## Abstract

Objectives: Use of off-label tissue graft materials, such as acellular dermal matrix (ADM), for in utero repair of severe spina bifida (SB), where primary skin layer closure is not possible, is associated with poor neurological outcomes. The cryopreserved human umbilical cord (HUC) patch has regenerative, anti-inflammatory, and anti-scarring properties, and provides watertight SB repair. We tested the hypothesis that the HUC is a superior skin patch to ADM for reducing inflammation at the repair site and preserving spinal cord function. Methods: In timed-pregnant ewes with twins, on gestational day (GD) 75, spina bifida was created without a myelotomy (functional model). On GD 95, repair was performed using HUC vs. ADM patches (randomly assigned) by suturing them to the skin edges. Additionally, full thickness skin closure as a primary skin closure (PSC) served as a positive control. Delivery was performed on GD 140, followed by blinded to treatment neurological assessments of the lambs using the Texas Spinal Cord Injury Scale (TSCIS) for gait, proprioception, and nociception. Lambs without spina bifida were used as controls (CTL). Ex vivo magnetic resonance imaging of spines at the repair site were performed, followed by quantitative pathological assessments. Histological assessments (blinded) included Masson’s trichrome, and immunofluorescence for myeloperoxidase (MPO; neutrophils) and for reactive astrocytes (inflammation) by co-staining vimentin and GFAP. Results: The combined hind limbs’ TSCIS was significantly higher in the HUC group than in ADM and PSC groups, *p* = 0.007. Both ADM and PSC groups exhibited loss of proprioception and mild to moderate ataxia compared to controls. MRI showed increased pathological findings in the PSC group when compared to the HUC group, *p* = 0.045. Histologically, the meningeal layer was thickened (inflammation) by 2–3 fold in ADM and PSC groups when compared to HUC and CTL groups, *p* = 0.01. There was lower MPO positive cells in the HUC group than in the ADM group, *p* = 0.018. Posterior column astrocyte activation was increased in ADM and PSC lambs compared to HUC lambs, *p* = 0.03. Conclusion: The HUC as a skin patch for in utero spina bifida repair preserves spinal cord function by reducing underlying inflammation when compared to ADM.

## 1. Introduction

Mid-gestation in utero spina bifida (SB) repair decreases co-morbidities, including shunt placement and ambulation when compared to postnatal repair [1,2]. Repair protects the spinal cord and prevents hindbrain herniation due to cerebral spinal fluid leakage [3,4]. Despite in utero repair, 58% of children were unable to ambulate independently and 27% required tethered cord surgery by school age [1,5]. Incomplete benefit is attributed to existing neurological injury before repair [6,7], suboptimal closure and inflammatory sequelae leading to spinal cord tethering and syringomyelia [8]. In utero repair consists of a deeper dural-myofascial layer and skin layer closures. In 80% of cases primary skin closure is possible, where the remainder requires tissue grafting [9]. Optimal tissue graft material for skin closure remains unknown. Acellular dermal matrix (ADM) has been used off-label in the pivotal management of myelomeningocele study when primary skin closure could not be achieved [1]. It is a decellularized matrix from human cadaveric skin [1,9]. The effects of ADM use for skin closure compared to primary skin closure remains unknown in terms of subsequent surgeries for tethered cord and the findings of syringomelia [10].

The cryopreserved human umbilical cord (HUC) has anti-inflammatory, anti-scarring, and regenerative properties, and is composed of a novel matrix called heavy chain hyaluronic acid/pentraxin3 (HC-HA/PTX3), which promotes regeneration of local native cells [11,12,13,14,15,16,17,18,19,20,21,22,23,24,25,26,27]. It has shown promising results for corneal and conjunctival surface reconstruction, and for dermal and orthopedic applications [23,24,25,26,27]. We evaluated the HUC for watertight in utero SB repair using a myelotomy (hindbrain herniation) sheep model and found that the skin had healed completely healed at the repair site along with hindbrain herniation reversal [28,29]. In a retinoic acid-induced SB rat model, the HUC patch lowered acute inflammation and cell death, and displayed organized cell growth compared to ADM [30]. In two human cases where the HUC was used as a skin patch, we found hindbrain herniation reversal, normal bladder control, and normal lower limb function [31].

Here, we compared clinical, radiological, and histological differences amongst defects closed with HUC vs. ADM skin patches in a surgically-created functional SB pregnant sheep model, generated without a myelotomy. This intact arachnoid layer model is well validated to test the prevention of ongoing damage to the exposed spinal cord [3,32].

## 2. Methods

The study protocol was approved by the Institutional Animal Care and Use Committee at UT Health School of Medicine in Houston (protocol #AWC-14-0177). All animal care and experimentation were performed in compliance with the Guide for the Care and Use of Laboratory Animals.

### 2.1. SB Model Creation

**Figure 1:**
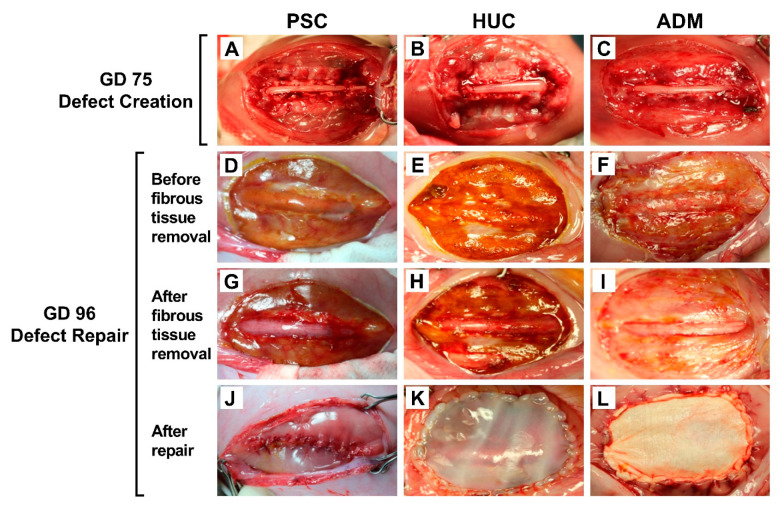
Creation and repair of spina bifida defects in sheep model fetuses. Representative images are depicted from the three SB surgical repair groups, with those being primary skin closure (PSC) and skin closure with either the cryopreserved human umbilical cord (HUC) or acellular matrix dermal (ADM) patches. (**A**–**C**) Creation of SB defects after skin, muscle, and posterior elements from the spinal canal and dura were removed, such that arachnoid layers were exposed. (**D**–**F**) Images of lesion sites immediately after lambs’ hysterotomies, showing fibrous tissue covering the lesions. (**G**–**I**) Images after fibrous tissue removal by microdissection to re-expose the meningeal layers and intact arachnoid layers. CSF can be appreciated beneath the meningeal and arachnoid layers. (**J**–**L**) Images showing repaired defects. (**J**) PSC was performed with full thickness mobilization of the skin, where subcutaneous tissue was sutured to the midline and then closed by primary closure. (**K**) The HUC patch was sutured circumferentially to the skin edges with its epithelial side facing the amniotic cavity and its mesenchymal surface facing the meningeal layer. (**L**) The ADM patch was sutured over the defect in a manner similar to that of the HUC patch.

Ultrasound-verified timed-pregnant sheep were obtained from K-Bar Livestock, L.L.C. (Bastrop, TX, USA) for the creation of the SB sheep model, as has been previously described [3,33]. Briefly, surgery was performed on gestational day 75 (term: 145 days) under general anesthesia in a supine position with a left lateral tilt. Under sterile conditions, a laparotomy was performed using a mid-line incision followed by a hysterotomy. As described previously [3,28,33], a SB defect was made surgically between the spinal levels L2 and L6 to create an approximate 4 cm × 5 cm defect. The paraspinous muscles and posterior lamina of vertebra were removed, and the subdural space entered using bone rongers, followed by dura removal and the remaining portion of the posterior lamina. This produced an exposed spinal cord with an intact arachnoid layer (Figure 1A–C). After the fetus was repositioned back within the amniotic cavity, the uterine incision was sutured in two layers using 2-O Vicryl (Ethicon Inc. Somerville, NJ, USA). Nafcillin (1 g; AuraMedics Pharma LLC, E. Windsor, NJ, USA) was infused into the amniotic cavity as an antibiotic prophylaxis. Once the uterus was placed back into the abdomen, the skin and fascial layers’ incisions were closed. 

### 2.2. SB Defect Repair

Repair surgeries were performed on gestational day 96. The sheep underwent general anesthesia followed by a laparotomy. Following the hysterotomy, a fibrous layer was noted to cover the site of the defect, which was removed using microsurgical instruments (Figure 1D–F). As a result, the arachnoid layer was re-exposed (Figure 1G–I). Fetuses that survived to gestational day 96 were randomly assigned to be repaired by PSC, or with a HUC (TissueTech^TM^ Inc., Miami, FL, USA) or ADM (AlloDerm^®^, BioHorizons, Birmingham, AL, USA) patch. For PSC group lambs, the full thickness of the skin edges was mobilized to the midline using a blunt dissection. The skin was closed using a 4-O Monocryl (Ethicon Inc. Somerville, NJ, USA) suture in a running lock manner (Figure 1J). For HUC or ADM patch repair lambs, a patch was trimmed to the size of the skin defect and sutured circumferentially to the skin edges using 4-O Monocryl (Ethicon Inc. Somerville, NJ, USA) in a running locking fashion (Figure 1K–L). The fetuses were repositioned within the amniotic cavity and the uterine wall closed. Four lambs without SB were considered negative controls and managed expectantly.

### 2.3. Delivery and Clinical Outcome Assessment

All fetuses were delivered between gestational day 139–142 by a planned cesarean delivery under general anesthesia. After delivery the fetuses were transitioned to room air by stimulation and drying. The ewes were euthanized immediately after cesarean delivery. After delivery, skin healing was assessed visually for complete, partial, or no healing based on keratinization and hair growth. The clinical assessment of hind limb function was performed by video recording during examination. Treatment masking per lamb was achieved by covering the lower spine of the lamb with a 10 cm × 10 cm bandage. Two independent examiners, blinded to the treatment assignment of lambs, reviewed the videos to assign Texas Spinal Cord Injury Scale (TSCIS) scores. Neurological assessments were performed two days after birth using the TSCIS as recently described by our group [29]. Briefly, the scale allows for a combined score of gait, proprioceptive positioning and nociception, allowing for a maximum score of 10 per limb [34,35,36,37]. Gait ambulation was assessed by tail walking, that is by holding the lamb upright by the tail or by using a sling near the lower spine area. For gait, scores between 0 to 6 per limb were assigned based on the presence and clinical significance of movement. A score of 0 would result when no voluntary movement was seen when supported. Gait would be assigned 1 if there was intact limb protraction with no ground clearance, 2 if intact limb protraction inconsistently cleared the ground, 3 if there was consistent limb protraction clearing the ground at least 75% of the time, 4 if ambulatory and consistently clearing the ground with moderate paresis-ataxia (will fall occasionally), 5 if ambulatory and consistently clearing the ground with mild paresis-ataxia (does not fall, even on a slick surface), and 6 if the lamb had normal gait. Proprioceptive positioning, also referred to as knuckling, was measured by a postural reaction test after placing the dorsum of the manus or pes on a non-sticky surface while the lamb’s ventrum was supported with one hand. It was scored as normal (score = 2) if they were able to correct the limb immediately. Lambs that replaced the hoof, but took >2 s to do so, or had difficulty doing so, were referred to as delayed and scored as 1. An absent response was scored as 0. Nociception was assessed by applying a painful stimulus to the limb and observing the lamb for physiological retraction or behavioral (orientation towards the stimulus, vocalization, licking) responses [35,36]. Superficial nociception (soft tissue pain) was tested by applying a hemostat to the inter-digital webbing [37]. If no superficial nociception was detected, deep nociception (bone or joint pain) was evaluated by cross-clamping a nail bed, digit or the distal limb with a hemostat [37]. Lambs were scored as having normal nociception (score = 2), no superficial nociception (score = 1) and no deep and superficial nociception (score = 0). The TSCISs were scored twice independently from video recordings. Euthanasia was performed under general anesthesia with a thoracotomy and cardioperfusion using 10% normal buffered formalin (NBF, Fisher Scientific, Hampton, NH, USA). The defect site was excised with a 3cm margin of tissue. The tissues were then further fixed in 10% NBF.

### 2.4. Magnetic Resonance Imaging and Analysis

Due to the risk of Q-fever exposure from live animal imaging of lambs [38], ex vivo of formalin fixed spines were imaged using magnetic resonance imaging (MRI), with well-established methods [39]. The data were acquired on a 7T/30 USR MRI scanner (Bruker BioSpin, Ettlingen, Germany) with a water-cooled shielded gradient coil system (Model BGA 12, 11 cm i.d.) The transmission and reception for scanner control was based on the vendor-supplied birdcage resonator with 72 mm i.d. using ParaVision software (PV 5.1, Bruker Biospin, Ettlingen, Germany). A pilot scan facilitated the placement of the spine in the magnet’s center. All following scans were acquired with a resolution of 0.16 × 0.16 × 0.5 mm (x,y,z) in axial orientation. In all scans, fat was suppressed. Anatomical images were acquired with T1-, T2-, and T2*-weighted protocols with the specifications listed in Supplemental Table S1. The spines were scanned in a container filled with nuclear magnetic resonance neutral Fomblin, a per-fluorinated oil (Solvay, Bollate, Italy), to reduce susceptibility artifacts and avoid the strong signal of hydrogen containing solvents [39].

MRI images were analyzed by two reviewers blinded to treatment allocation. The lesion site was identified by the anatomically absent posterior elements of the spine. MRI images were evaluated for anatomy and graded for lost gray and white matter differentiation with or without distortion as sequela of ongoing inflammatory insult. Grade 1—clear distinction of gray and white matter; Grade 2—moderate distortion (border between grey and white matter is unclear, but less than 100%); and Grade 3—severe distortion (distinction between grey and white matter is unclear 100% of cross section). The percentage of slices per different grade of differentiation were quantified and compared. Additionally, slices were interrogated for cystic lesions and hemorrhages, which were confirmed by histology, followed by quantitative assessment of the lesion’s proportion for those anomalies.

### 2.5. Histology

Following MRI scan, the spines were sectioned at 5 intervertebral disc levels, including above lesion (L1–L2), high-lesion (L2–3), mid-lesion (L3–4), low-lesion (L4–L5), and below the lesion (L6–S1), for primary tissue analysis. Additional sections were obtained in 5 mm increments for each vertebral level to confirm radiological findings histologically. Tissue were paraffin-embedded and cut at 4 μm thicknesses. Paraffin-embedded formalin (Fisher Scientific, Hampton, NH, USA) fixed sections were deparaffinized using 100% xylene (VWR, Radnor, PA, USA) and rehydrated in serial dilutions of ethanol (100%, 95%, 70%, and 50%, respectively; Decon, King of Prussia, PA, USA). Hematoxylin and Eosin (H&E; Merck KGaA, Darmstadt, Germany) and Masson’s Trichrome (Diagnostic BioSystems, Pleasanton, CA, USA) staining were performed to assess wound and skin healing capabilities, spinal cord and meninges’ anatomies, and the arachnoid layer’s average thickness per level. Arachnoid layer collagen architecture was assessed using Picrosirius red staining (Abcam, Cambridge, MA, USA). The stained slides were viewed under circular polarized light to assess collagen birefringence, which represents an index of the organization and orientation of collagen fibers. Nissl staining was performed to quantify anterior horn cells using Cresyl Violet Acetate Working Solution (Electron Microscopy Sciences, Hatfield, PA, USA). Images were acquired using a Zeiss Axio Imager.A2 microscope (Carl Zeiss AG, Oberkochen, Germany) equipped with X-cite 120LEDmini system and Axiocam 506 color camera (Pixel size; 4.54 × 4.54 µm, 14 bit). Optical sections were obtained by using a 10× (N.A. = 0.3, EC Plan Neofluar) or a 20× (N.A. = 0.5, EC Plan Neofluar) objective. Individual images were collected and processed using ZEN (Blue edition, Carl Zeiss AG, Oberkochen, Germany).

### 2.6. Immunofluorescence

Following de-paraffinization, antigen retrieval was performed using a 10 mM sodium citrate buffer (pH 6.0) at 95 °C for 10 m then allowed to cool in the buffer for 20 min, or proteinase-K antigen retrieval solution of pH 8.0, as was appropriate for the primary antibody followed by washing the slides for 5 min in phosphate-buffered saline (PBS). To block non-specific binding after antigen retrieval, the slides were incubated with PBS containing 5% goat serum and 0.2% Triton-X-100 for 1hr at room temperature. Slides were washed with PBS briefly and incubated overnight with primary antibody at 4 °C. Primary antibodies were used to assess skin regeneration using anti-keratin 1 (1:100; Abcam, Cambridge, MA, USA), anti-keratin 5 [40] (1:100; Millipore, Burlington, MA, USA), acute inflammatory response to patch using anti-myeloperoxidase (MPO; 1:100; Thermo Scientific, Waltham, MA, USA) for neutrophils, the myofibroblast activation in the arachnoid layer using anti-α-smooth muscle actin (α-SMA; 1:200, Agilent Technologies, Santa Clara, CA, USA). Additionally, the spinal cord inflammation was quantified using markers for reactive astrocytes by co-localization of anti-glial fibrillary acidic protein (GFAP; 1:200; Abcam, Cambridge, UK), anti-vimentin (1:100; Sigma, St. Louis, MO, USA). Immunofluorescence detection was obtained using Alexa Fluor 488 goat anti-mouse or Alexa Fluor 568 goat anti-rabbit antibodies (1:1000; Invitrogen, Carlsbad, CA, USA), with DAPI (ThermoFisher Scientific, Carlsbad, CA, USA) counterstaining nuclei. The primary antibody omitted stains served as negative controls (data not shown). Images were acquired using a Zeiss Axio Imager.A2 microscope (Carl Zeiss AG, Oberkochen, Germany) equipped with X-cite 120LEDmini system and Axiocam 506 color camera (Pixel size; 4.54 × 4.54 µm, 14 bit). Optical sections were obtained by using a 40× objective (N.A. = 0.75, EC Plan Neofluar). Fluorescence excited with 470 ± 40 nm, 546 ± 12 nm or 365 nm and emission monitored 525 ± 50 nm, 575−640 nm, or 445 ± 50 nm, respectively. Individual images were collected and processed using ZEN (Blue edition, Carl Zeiss AG, Oberkochen, Germany). For each tissue section, all histological analyses were performed, and cell counts were obtained in a blinded manner by two separate observers, with discrepancies resolved by a third more senior reviewer.

## 3. Statistics

The data are presented as descriptive statistics. Inferential statistics were performed using ANOVA for normally distributed data and Kruskal–Wallis test for non-normally distributed data to compare differences amongst the groups. For categorical outcomes, a chi-squared test was performed intra-observer variability of TSCIS scores for gait and proprioception were calculated using Bland–Altman bias and agreement analysis (Supplementary Figure S2). Prism 9.1 (GraphPad Software, Inc., La Jolla, CA, USA) was used for analysis, where a *p*-value of <0.05 was considered significant.

## 4. Results

The study was performed in 17 pregnant ewes with 31 fetuses (singleton pregnancy, *n* = 2; twin pregnancies *n* = 13; and a triplet pregnancy). Twenty-seven fetuses underwent surgical creation of SB defects, where 6 (22%) had a fetal demise before the repair stage. A total of 21 fetuses were randomly assigned to three treatment groups: 7 were PSC repaired (male: 2), 8 had HUC patch repair (male: 5), and 6 had ADM patch (male: 3) repair of their defects. Abdominal incision dehiscence occurred in a ewe with twins after having PSC performed on one of the fetuses and a HUC patch based-repair on the other, and were therefore euthanized. Lambs transitioned to room air at birth without any difficulties, excepting for a lamb in the HUC repair group that demised within 1 h after delivery for an unknown reason. Thus, the final lambs for assessment were 6 in the PSC group, 6 in the HUC patch repair group, and 6 in the ADM patch repair group. Four lambs having no SB defects served as negative controls. All lambs showed complete spontaneous voiding, which was confirmed ultrasonically by post-void residual volume. Additionally, all lambs demonstrated normal tail movement.

Preserved spinal cord function in the HUC repair group compared to the PSC and ADM repair groups.

**Figure 2:**
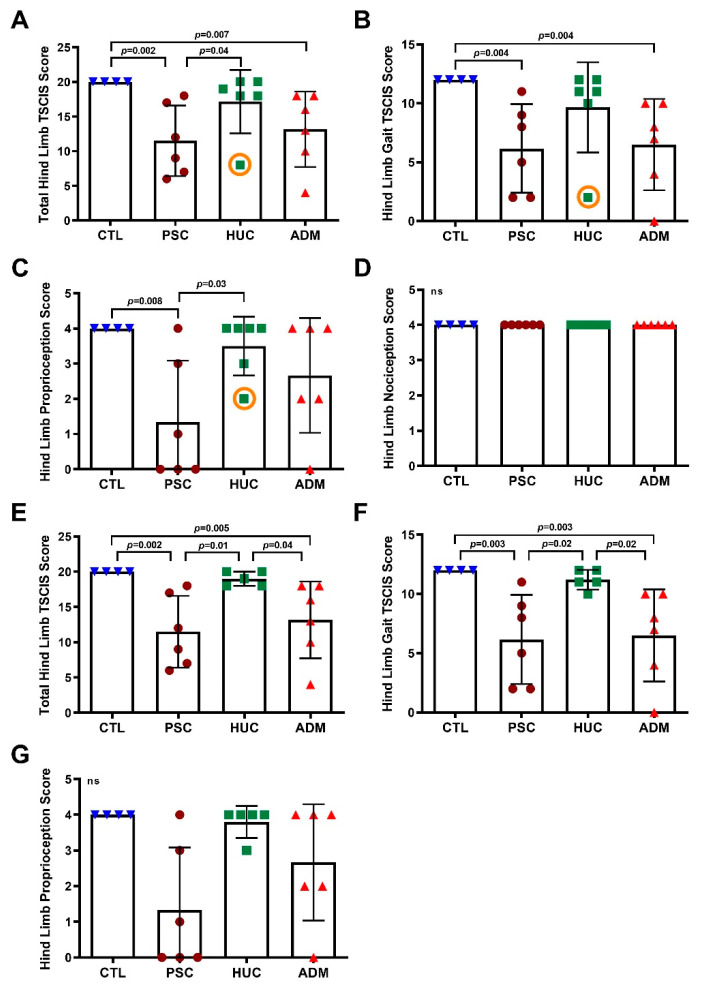
Texas Spinal Cord Injury Scale (TSCIS) scores of spina bifida defect repaired lambs. TSCIS scores for control lambs and the three SB surgical repair groups are provided. Graphs (**A**) through (**D**) include data from a lamb that was repaired with a HUC patch observed to have the lowest score, which was later found to have had a fresh hemorrhage on MRI and histology, whereas Graphs (**E**) through (**G**) does not. (**A**) Total TSCIS scores for control (CTL), PSC, HUC, and ADM repaired groups are given. (**B**) Hind limb gait TSCIS scores for CTL and repair groups are shown, with those being the three surgically repaired groups. (**C**) Analysis of hind limb TSCIS proprioception scores. (**D**) Hind Limb nociception for all evaluable lambs in the study are provided. (**E**) Post hoc analysis of total TSCIS scores between all repair and control groups with the aforementioned HUC outlier removed. (**F**) Post hoc analysis of hind limb gait TSCIS scores between all repair and control groups with the HUC lamb having the lowest score, as described, removed. (**G**) Post hoc analysis of hind limb proprioception TSCIS scores between all repair and control groups with the HUC lamb having the lowest score, as described, removed. Data are shown as mean ± s.d. *p*-values were determined by ANOVA, Kruskal–Wallis test, Two-stage linear step-up procedure of Benjamini, Krieger, and Yekutieli, whereby *p* < 0.05 were considered significant and ns represents non-significant (*p* > 0.05) data.

Forelimb assessments were normal in all lambs (data not shown). For hind limb assessment, the normal control lambs were assigned the maximal total TSCIS score of 20 (12:Gait + 4:proprioception+ 4:nociception). Hind limb assessment showed no differences between control and HUC-repaired lambs for overall hind limb function, and individually in their gait, proprioception and nociception scores (Figure 2A–D). Lambs repaired with PSC or with the ADM patch achieved lower total scores to the control lambs (Figure 2A). The lambs that were repaired with PSC had lower total scores than the HUC-repaired lambs (Figure 2A). Total TSCIS scores were higher in the HUC repair group compared to the ADM repair group, however the data did not reach statistical significance (Figure 2A; *p* = 0.11). On further analysis of components of TSCIS, hind limb gait scores were significantly lower in the PSC and ADM compared to controls, and no other differences were significant (Figure 2B). Proprioception was significantly lower in the PSC group compared to CTL and HUC-repaired lambs, and no differences were found in other groups (Figure 2C). Nociception scores were similar between all groups evaluated (Figure 2D). The lamb with the lowest score in the HUC repair group (marked by an orange circle in Figure 2A–C), experienced a trauma at delivery and upon resuscitation, where the lesion site had a blunt impact. Upon euthanasia and excision of the defect site, it was noted that the aforementioned lamb had a fresh hemorrhage around the spinal cord, indicating that the impact that occurred immediately after delivery resulted in the sequelae of abnormal behavior and spinal cord function impairment. Upon further analysis following the clinical exclusion of the aforementioned lamb we found that the HUC repair group had a higher overall TSCIS and gait score than the ADM repair group (Figure 2E–G). The main contributor to the lower scores in the PSC and ADM repair groups were attributed to loss of proprioception and ataxia (Figure 2E–G).

### 4.1. Delayed Epithelialization of the ADM Together with Neutrophil Accumulation

**Figure 3:**
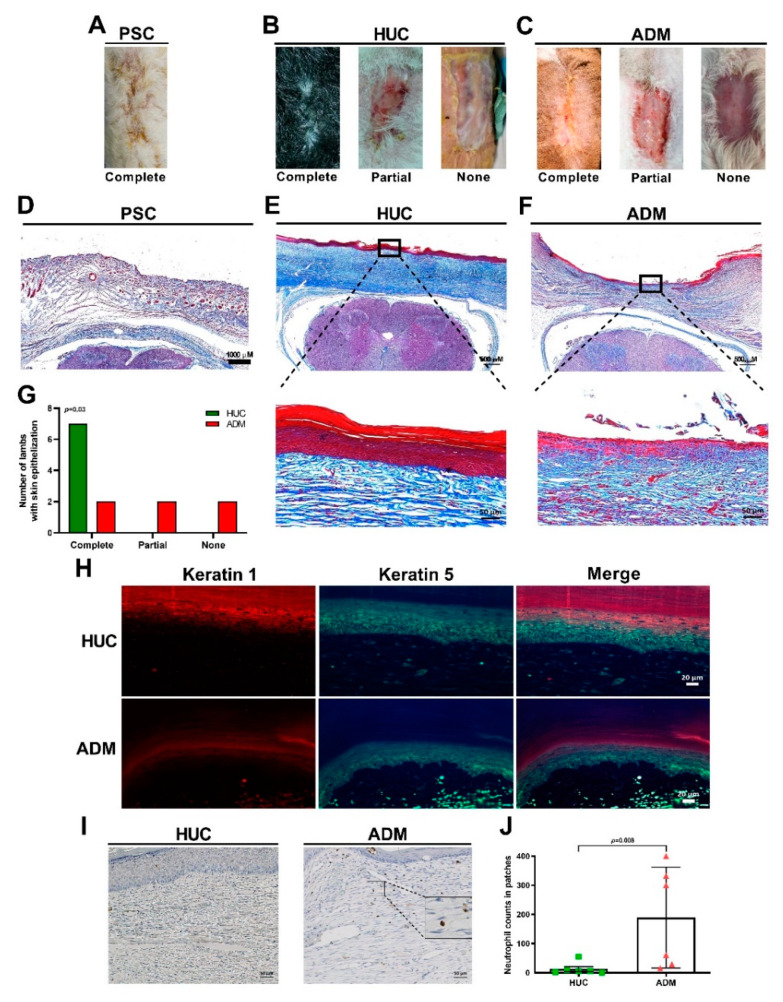
The ADM patch accumulates neutrophils and or monocytes with delayed and disorganized skin epithelialization. (**A**) PSC lambs with SB defects results in complete wound healing. (**B**) Use of the HUC patch during SB repair leads to complete, partial or unrepaired defect sites. (**C**) ADM patch use to repair SB defects in utero causes lambs to be born with variable wound healing capabilities, with those being complete, partial or remaining completely unrepaired at defect sites. (**D**) Representative image of a PSC group mid-level sagittal section of the defect repair site using Masson’s trichrome staining (1000 µM). (**E**) A representative mid-level sagittal section image of a HUC patch repaired defect site by Masson’s trichrome staining (500 µM). A 50 µM magnified image of the skin at the lesion is shown in the lower panel. (**F**) A representative mid-level sagittal section image of a ADM patch repaired defect site by Masson’s trichrome staining (500 µM). A 50 µM magnified image of the skin at the lesion is shown in the lower panel. (**G**) Skin epithelialization occurred in all lambs repaired with the HUC patch. (**H**) Immunofluorescent anti-keratin 1 and anti-keratin 5 co-staining of defects repaired using the HUC patch and ADM the patch. (**I**) MPO staining of HUC and ADM patches at defect repair sites. (**J**) Quantification of the number of neutrophils and or monocytes infiltration, that being MPO positive cells, into HUC and ADM patches at defect repair sites.

Visual examination of the repair sites at birth showed that lambs repaired conventionally had completely healed skin (Figure 3A). In the HUC repair group, we found that the skin was completely healed in 1 lamb, 5 lambs were partially healed, and no healing occurred in one of the lambs (Figure 3B). When ADM-repaired lambs were assessed, it was noted that 2 lambs’ defect sites were completely healed, 3 lambs had partial healing, and that 1 lamb’s defect did not heal (Figure 3C). Masson’s Trichrome analysis of the cross-sectioned defect repair sites was used to investigate the degree of epithelialization at repair sites. This revealed that the skin in the PSC group had epithelialized and healed (Figure 3D), and that the HUC (Figure 3E) repaired lambs had a greater degree of epithelial organization and growth than the ADM-repaired lambs at their defect repair sites (Figure 3F). Although not all HUC-repaired lambs had completely repaired defect sites at birth, all HUC patches promoted skin epithelialization compared to lower epithelialization associated with ADM patch use (Figure 3G). The thickness of the repair site in midline measured no difference between HUC group (946 µm ± 424 µm) to ADM group (956 µm ± 264 µm; *p* = 0.9). However, the PSC group (1548 µm ± 348 µm) was significantly thicker than HUC (*p* = 0.02) and ADM groups (*p* = 0.03). Epithelial cell differentiation and proliferation, evaluated by keratin 1 and keratin 5 immunofluorescence [40], respectively, revealed normal polarization in both HUC- and ADM-repaired lambs (Figure 3H). Furthermore, neutrophils and or monocytes were recruited to the ADM patch and not to the HUC patch (Figure 3I–J). Taken together, these results indicate that the HUC patch’s attributes of promoting organized wound healing and a reduction in graft-associated inflammation is superior to that of the ADM patch for the repair of SB defects.

### 4.2. Radiological Evidence of Increased Spinal Cord Abnormalities in the PSC and ADM Repair Groups Compared to the HUC Repair Group

**Figure 4:**
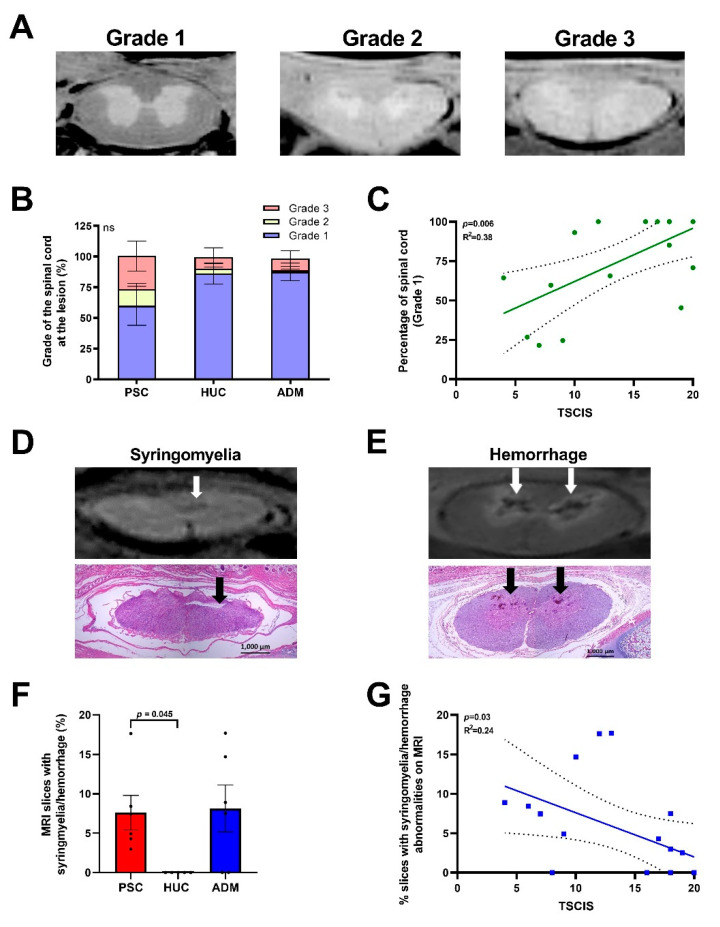
Radiological findings in terms of neurological outcomes. MRI evidence of lower pathology grades with increased TSCIS scores, and the presence of syringomyelia affecting neurological outcomes negatively. (**A**) Representative images of MRIs graded as either Grade 1 (slight distortion), Grade 2 (moderate distortion), or Grade 3 (severe distortion) by capability of distinguishing gray from white matter. (**B**) Grade of the lesion for all defect repair groups are provided as a percentage. (**C**) Simple linear regression of the percentage of Grade 1 per spinal cord is shown as a function of the TSCIS score. Confidence intervals at 95% are provided. A *p* = 0.05 was considered significant. (**D**) MRI showing fluid collection at the central canal (white arrow) and the corresponding H&E image showing syringomyelia (black arrow) with lining extending into the central canal. (**E**) Diffuse hemorrhage seen in a spinal cord on MRI (white arrow) was confirmed using H&E histology (white arrow). (**F**) Quantitative comparison of syringomyelia/hemorrhage for the percentage of sections per lamb on MRI noted in the spinal cords. (**G**) Association of the percentage of slices with syringomyelia/hemorrhage and TSCIS score as seen on H&E sections were analyzed by simple linear regression using 95% confidence intervals, where *p* = 0.05 was considered significant. *p*-values for continuous data were determined by ANOVA, Kruskal–Wallis test using Dunn’s multiple comparisons test.

Serial MRI images of the lambs’ spinal cords at defect sites were obtained according to instrumentation settings (Supplemental Table S1). Assessments of MRI images showed different grades of grey and white matter differentiation. Grade 1––clear distinction of gray and white matter; Grade 2––moderate differentiation (border is unclear, but less than 100%); and Grade 3––severe (distinction is unclear, 100% of cross section) (Figure 4A). Higher grades of differentiation loss were noted in PSC, but did not reach statistical significance (Figure 4B). The percentage of Grade 1 slices in MRIs directly correlated with TSCIS scores (Figure 4C). Furthermore, syringomyelia (Figure 4D) and hemorrhages (Figure 4E) were significantly higher in the PSC group compared to HUC repaired lambs, and there were no significant differences between other groups (Figure 4F). The presence of syringomyelia and hemorrhages were confirmed histologically in H&E sections (Figure 4D,E; Supplemental Figure S1). The percentage of syringomyelia/hemorrhage were inversely correlated with TSCIS score (Figure 4G).

### 4.3. Meningeal Layer Thickness and Myofibroblasts in Outer Arachnoid Layer Differences

**Figure 5:**
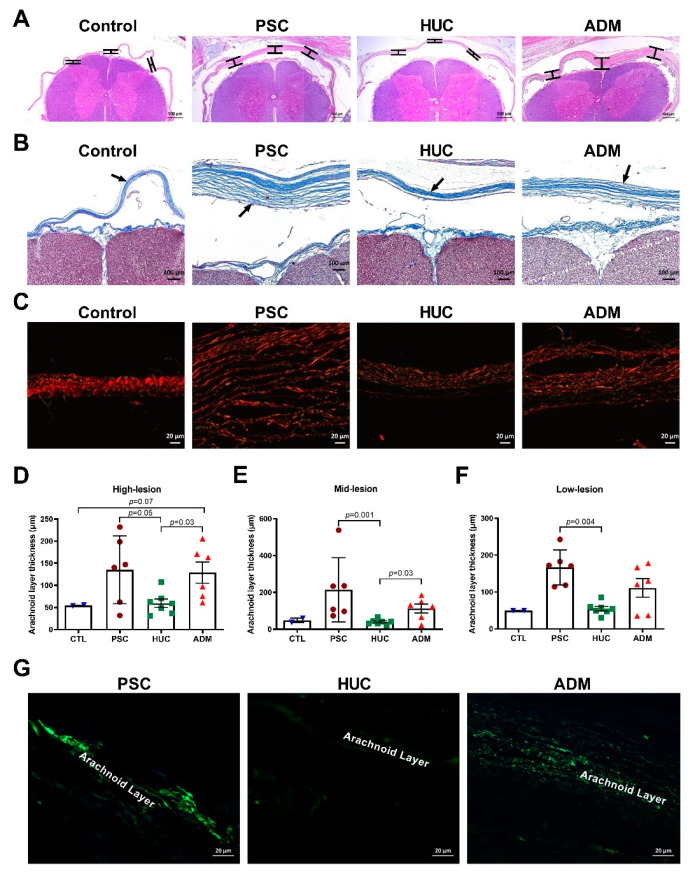
Arachnoid layer thickness and myofibroblast infiltration is greater when lamb spinal cord defects are repaired conventionally or with an ADM patch. Immunohistochemistry using (**A**) H&E, measurements were performed at three sites for each level as marked by black line segments (**B**) Masson’s trichrome showing the collagen fibers in the arachnoid layer, and (**C**) Picrosirius red immunofluorescent staining of defect repair sites for control lambs, the PSC group, and the HUC and ADM patch repair groups are shown. Arrows indicate arachnoid layers in the images. (**D**) Serial sagittal sections of the spinal cord at defect sites were stained by immunohistochemistry using Masson’s Trichrome, where (**D**) high-lesion values represent the arachnoid layer thickness towards the outer skin layer, (**E**) mid-lesion values, and (**F**) low-lesion values for arachnoid layer thickness toward the pia mater for control, PSC, HUC, and ADM groups. Data are shown as mean ± s.e.m. *p*-values were determined by ANOVA, Kruskal–Wallis test, two-stage linear step-up procedure of Benjamini, Krieger, and Yekutieli. (**G**) α-SMA immunofluorescent staining of mid-lesion arachnoid layers per defect repair group for myofibroblasts.

Inflammatory arachnoiditis is associated with arachnoid layer thickening with fibrosis [41,42]. Evaluation of 3 lesion levels by H&E (Figure 5A) and Masson trichrome staining (Figure 5B) identified blue collagen fibril deposition in arachnoid layers. At each level, the average of the three measurements were taken; one at the midline and two poster laterally where the posterior horn ends. Each level was further analyzed by Picrosirius red stain to understand the density and organization of collagen fibers (Figure 5C). The arachnoid layer was thickened in the PSC and ADM repair groups when compared with control or HUC-repaired lambs (Figure 5A–C). On quantitative assessment, there was no difference in arachnoid layer thickness between control and HUC lambs, and the PSC lambs had increased arachnoid layer thickness than the HUC repaired lambs at all three lesion levels (Figure 5D–F). There was increased thickness in the arachnoid layers in the ADM group compared to HUC repair at high and mid-lesion levels (Figure 5D,E). We hypothesized that thicker arachnoid layers may be a result of the contribution of fibrous tissue deposition. Mid-lesion level staining for α-SMA, a protein found transiently in myofibroblasts during normal tissue repair and chronic fibrotic pathologies [43], was enhanced in PSC and ADM defect repair groups compared with the HUC repair group (Figure 5G). We found that α-SMA positivity occurred in arachnoid layers for all lambs in the PSC (6/6) and ADM repair (6/6) groups as opposed to α-SMA positivity being found in a single section of a HUC repair lamb (1/7; *p* < 0.0001). Collectively, these data suggest that potentially fibrotic and thickened arachnoid layers as a sequela of ongoing inflammation at repair sites may be reduced through use of the HUC patch. In addition, in all levels of histology at the repair site, there was cerebrospinal fluid space noted between the posterior column and arachnoid layer suggesting no evidence of spinal cord tethering.

### 4.4. Reactive Astrocyte Enhancement in the PSC and ADM Repair Groups versus the HUC Repair Group

**Figure 6:**
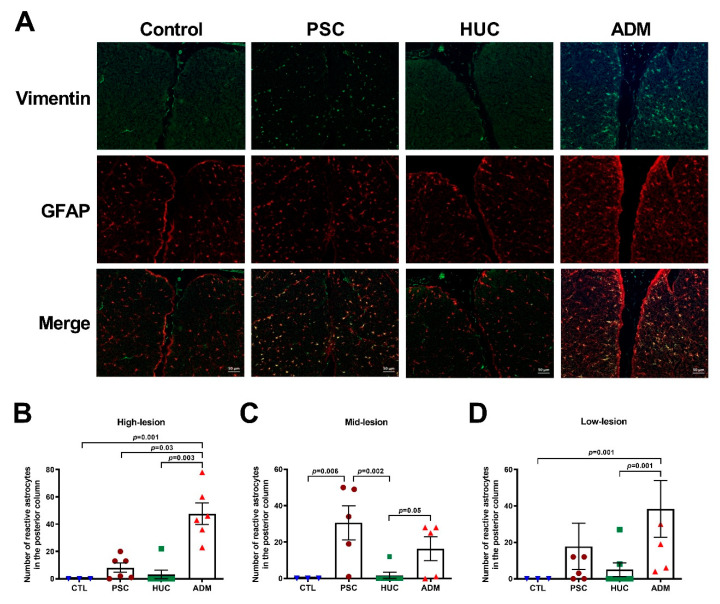
Reactive astrocytes are lower in spinal cord posterior columns of the HUC patch at defect sites than for PSC and HUC patch repair groups. (**A**) Immunofluorescent detection of reactive astrocytes in spinal cord posterior columns of control lambs and in PSC, HUC, and ADM patch defect repair groups by staining for vimentin and anti-glial fibrillary acidic protein (GFAP). (**B**) Quantification of the number of reactive astrocytes present in high-lesion tissue sections at defect repair sites. (**C**) Quantification of the number of reactive astrocytes present in mid-lesion tissue sections at defect repair sites. (**D**) Quantification of the number of reactive astrocytes present in low-lesion tissue sections at defect repair sites. All graphical data are shown as mean ± s.e.m. *p*-values were determined by ANOVA, Kruskal–Wallis test using the two-stage linear step-up procedure of Benjamini, Krieger, and Yekutieli.

Reactive astrocyte staining of tissue sections by GFAP and vimentin co-localization [44] indicated a generalized activity increase in spinal cord posterior columns for both PSC and ADM repair groups when contrasted with control and HUC-repaired lambs (Figure 6A). At the high-lesion level, prominent differences in astrocytic reactivity were identified between the ADM repair group and all other repair groups (Figure 6B). At mid-level defect repair site sections of spinal cords, both PSC and ADM groups had enhanced astrocyte activity than the HUC group, and PSC lambs had greater numbers of astrocyte reactivity than control or HUC-repaired lambs (Figure 6C). At lower-lesion site, astrocyte reactivity was greater in ADM-repaired lambs compared to control and HUC-repaired lambs (Figure 6D–F). Taken together, a prolonged neuroinflammatory insult may be possible with PSC and ADM patch use to repair SB spinal cord defects, and that this phenotype is not extended to HUC patch-based repair. No differences were found in the numbers of anterior horn cells at mid-level sections of defect repair sites amongst evaluable groups (Supplemental Figure S2A–D).

## 5. Discussion

We found that using the HUC patch for in utero repair of SB skin defects improves spinal cord function through reducing inflammation at repair sites, and by preserving spinal cord anatomy when compared to ADM use in this functional sheep model. The benefits noted in the HUC patch group are attributed to lowering inflammation at the repair site, rapid epithelization, a decreased arachnoid layer thickness, and a reduction in spinal cord syringomyelia and hemorrhagic events.

We show that arachnoid layer thickening with myofibroblast (scar forming cell) activation, particularly notable in the ADM and PSC groups, can been explained by chronic inflammation and scar tissue formation. The lack of such findings in the HUC group is probably secondary to cellular signaling components within the graft, which may have dedifferentiated myofibroblasts into native fibroblasts by activating BMP signaling [20,27]. It has been found that umbilical cord extracts have revert myofibroblasts to their naïve phenotype via BMP signaling activation [20,27]. Recent data suggests that this HC-HA/PTX3 complex can directly modulate adaptive immunity by increasing polymorphonuclear cell apoptosis, by promoting phagocytosis of apoptotic cells by M2 macrophages, and by suppressing Th1/Th17 lymphocytes [22,23]. The presence of syringomyelia in ADM-repaired lambs and the PSC lambs are similar to what is commonly found in human in utero SB repair cases [1,45]. The development of syringomyelia at the repair site could be related to altered CSF traffic related to arachnoid fibrosis, and to inflammation surrounding the spinal cord, as is noted in the ADM repair and PSC groups. Any process that increases the spinal cord’s subarachnoid space’s pressure or compression may cause an obstruction of CSF flow, as would inflammation or tethering, thereby producing a pressure differential across the obstructed segment and resulting in syringomyelia [46,47,48].

Generalized inflammation and delayed repair, as was evident in the ADM patch, may explain the reduction in spinal cord function preservation. The translational findings could have a significant impact on human spina bifida repair, especially in developmental and functional outcomes. During human in utero SB repair, the arachnoid layer is typically disrupted or damaged, resulting in scar formation between the repair site and spinal cord [5,6]. This scarring effect, otherwise known as spinal cord tethering, is also noted in children undergoing postnatal SB defect repair [8]. The non-myelotomy SB model that was used in this study does not completely reproduce the human defect, where there is a disruption of the spinal cord. The PSC treatment arm, a well-established surgical model [49,50], was used as a positive control for the experiments. Repair in humans typically makes use of a myofascial flap, thereby possibly resulting in different functional outcomes, which has not been studied in a sheep model to date. It is possible, however, that the creation and suturing of a myofascial flap may generate additional tissue injury, and in doing so increase scarring and tethering. The favorable effects of reducing inflammation and scar formation overall, as was found in the HUC treatment group, should benefit the underlying layers. The HUC patch is readily available for purchase, however regulatory authorities are still evaluating its feasibility and efficacy in the clinical setting.

The creation of the SB defect in sheep without a myelotomy at mid-gestation was performed similarly to methods originally described by Meuli et al. [3], which was used to evaluate spinal cord function [32]. Additionally, fibrous tissue removal by fine dissection to re-expose the arachnoid layer during repair surgery was used in an analogous manner to Wang et al. [51]. The removed fibrous tissue was found to be composed of disorganized collagen filled with myofibroblasts (Supplemental Figure S3). The direct exposure of the arachnoid layer to the repair site provided a more comparable physiological state to that of human defects, compared to if the fibrous tissue had been left in place, where it often mistaken for new dura mater regeneration [52]. Unlike Mueli et al. [3], we did not perform myofascial flap rotation from the latissimus dorsi. It remains to be determined whether the presence of a myofascial flap that covers an intact arachnoid layer beneath the skin closure may yield better results than without the myofascial flap. Hence, our PSC group may not be comparable to the conventional repair method used in the Mueli et al. [3] The lack of complete healing of the patch noted in the lambs is similar to results obtained in humans when they are repaired with a patch [31].

The major strengths of the study included randomization and investigator blinding, such that treatment allocation and assessment bias was reduced when evaluating histological and functional outcomes. In addition to hypothesis testing, possible mechanisms for the functional outcomes were evaluated. To our knowledge, there are no rigorously designed comparative studies evaluating different allograft materials to use for severe spina bifida defect where primary skin closure was not possible. However, the surgical model of SB, inherently having an anatomically abnormal spinal cord with dysraphism of the neural placode when compared to the human spinal cord with deficient neural elements in the posterior part of the spinal cord. We were unable to evaluate the exact mechanism for lack of function through effects on the spinal cord tracts due to preservation techniques using formalin to reduce Q-fever exposure used in the study. Further, the meninges, including the arachnoid and pia mater, are disrupted in human SB cases, which thereby exposes neural elements and ependymal layers. This contrasts with the non-myelotomy model, where the arachnoid and pia mater are both intact, providing additional protection to the inner elements of the spinal cord. With the intact arachnoid layer in the non-myelotomy model, we were unable to evaluate the tethering. Thus, future studies are required to evaluate spinal cord tethering where the arachnoid layer will need to be removed [51,53,54].

In summary, we found that the HUC patch induces less inflammation and reduces neurological sequelae compared to ADM patch-based repair of in utero SB repair in a functional sheep model without a myelotomy. Future studies are required to compare the clinical outcomes in humans using different graft materials for skin closure.

## Supplementary Materials

The following are available online at https://www.mdpi.com/article/10.3390/jcm10214928/s1, Figure S1: Five level histology of the spine to assess for syringomyelia and hemorrhage, Figure S2: Anterior horn cell numbers are equally distributed amongst all defect repair sites of different repair methodologies, Figure S3: Fibrous tissue removed at defect repair sites prior to repair during surgery is scar tissue, Table S1: MRI Settings for the acquisition of lambs’ spinal cords at defect repair sites.

## References

[1] Adzick N.S., Thom E.A., Spong C.Y., Brock J.W., Burrows P.K., Johnson M.P., Howell L.J., Farrell J.A., Dabrowiak M.E., Sutton L.N. (2011). A randomized trial of prenatal versus postnatal repair of myelomeningocele. N. Engl. J. Med..

[2] Williams H. (2008). A unifying hypothesis for hydrocephalus, Chiari malformation, syringomyelia, anencephaly and spina bifida. Cereb. Fluid Res..

[3] Meuli M., Meuli-Simmen C., Hutchins G.M., Yingling C.D., Hoffman K.M., Harrison M.R., Adzick N.S. (1995). In utero surgery rescues neurological function at birth in sheep with spina bifida. Nat. Med..

[4] Bouchard S., Davey M.G., Rintoul N.E., Walsh D.S., Rorke L.B., Adzick N.S. (2003). Correction of hindbrain herniation and anatomy of the vermis after in utero repair of myelomeningocele in sheep. J. Pediatric Surg..

[5] Houtrow A.J., Thom E.A., Fletcher J.M., Burrows P.K., Adzick N.S., Thomas N.H., Brock J.W., Cooper T., Lee H., Bilaniuk L. (2020). Prenatal repair of myelomeningocele and school-age functional outcomes. Pediatrics.

[6] Farmer D.L., Thom E.A., Brock J.W., Burrows P.K., Johnson M.P., Howell L.J., Farrell J.A., Gupta N., Adzick N.S. (2018). The management of myelomeningocele study: Full cohort 30-month pediatric outcomes. Am. J. Obstet. Gynecol..

[7] Ben Miled S., Loeuillet L., Duong Van Huyen J.P., Bessieres B., Sekour A., Leroy B., Tantau J., Adle-Biassette H., Salhi H., Bonnière-Darcy M. (2020). Severe and progressive neuronal loss in myelomeningocele begins before 16 weeks of pregnancy. Am. J. Obstet. Gynecol..

[8] Mehta V.A., Bettegowda C., Ahmadi S.A., Berenberg P., Thomale U.W., Haberl E.J., Jallo G.I., Ahn E.S. (2010). Spinal cord tethering following myelomeningocele repair. J. Neurosurg. Pediatrics.

[9] Moldenhauer J.S., Soni S., Rintoul N.E., Spinner S.S., Khalek N., Martinez-Poyer J., Flake A.W., Hedrick H.L., Peranteau W.H., Rendon N. (2015). Fetal myelomeningocele repair: The post-MOMS experience at the Children’s Hospital of Philadelphia. Fetal Diagn. Ther..

[10] Papanna R., Bahtiyar O., Bennett K.A., Emery S., Lillegard J.B., Goldstein R., Goodnight W., Jatres J., Lim F.-Y., McCullough L.B. (2019). 229: Use of tissue grafts for in-utero spina bifida closure of large skin defects. Am. J. Obstet. Gynecol..

[11] Liu J., Sheha H., Fu Y., Liang L., Tseng S.C. (2010). Update on amniotic membrane transplantation. Expert Rev. Ophthalmol..

[12] Acharya G., Pavlovic M., Ewing L., Nollmann D., Leshko J., Huhta J.C. (2008). Comparison between pulsed-wave Doppler- and tissue Doppler-derived Tei indices in fetuses with and without congenital heart disease. Ultrasound Obstet. Gynecol..

[13] Cooke M., Tan E.K., Mandrycky C., He H., O’Connell J., Tseng S.C. (2014). Comparison of cryopreserved amniotic membrane and umbilical cord tissue with dehydrated amniotic membrane/chorion tissue. J. Wound Care.

[14] Tan E.K., Cooke M., Mandrycky C., Mahabole M., He H., O’ Connell J., McDevitt T.C., Tseng S.C.G. (2014). Structural and biological comparison of cryopreserved and fresh amniotic membrane tissues. J. Biomater. Tissue Eng..

[15] He H., Li W., Tseng D.Y., Zhang S., Chen S.Y., Day A.J., Tseng S.C. (2009). Biochemical characterization and function of complexes formed by hyaluronan and the heavy chains of inter-alpha-inhibitor (HC*HA) purified from extracts of human amniotic membrane. J. Biol. Chem..

[16] Zhang S., He H., Day A.J., Tseng S.C. (2012). Constitutive expression of inter-alpha-inhibitor (IalphaI) family proteins and tumor necrosis factor-stimulated gene-6 (TSG-6) by human amniotic membrane epithelial and stromal cells supporting formation of the heavy chain-hyaluronan (HC-HA) complex. J. Biol. Chem..

[17] He H., Zhang S., Tighe S., Son J., Tseng S.C. (2013). Immobilized heavy chain-hyaluronic acid polarizes lipopolysaccharide-activated macrophages toward M2 phenotype. J. Biol. Chem..

[18] Zhang S., Zhu Y.T., Chen S.Y., He H., Tseng S.C. (2014). Constitutive expression of pentraxin 3 (PTX3) protein by human amniotic membrane cells leads to formation of the heavy chain (HC)-hyaluronan (HA)-PTX3 complex. J. Biol. Chem..

[19] Tseng S.C.G., Espana E.M., Kawakita T., Di Pascuale M.A., Li W., He H., Liu T.-S., Cho T.-H., Gao Y.-Y., Yeh L.-K. (2004). How does amniotic membrane work?. Ocul. Surf..

[20] Li W., He H., Chen Y.T., Hayashida Y., Tseng S.C. (2008). Reversal of myofibroblasts by amniotic membrane stromal extract. J. Cell. Physiol..

[21] Guo P., Zhang S.Z., He H., Zhu Y.T., Tseng S.C. (2012). PTX3 controls activation of matrix metalloproteinase 1 and apoptosis in conjunctivochalasis fibroblasts. Investig. Ophthalmol. Vis. Sci..

[22] Chen S.Y., Han B., Zhu Y.T., Mahabole M., Huang J., Beebe D.C., Tseng S.C.G. (2015). HC-HA/PTX3 purified from amniotic membrane promotes bmp signaling in limbal niche cells to maintain quiescence of limbal epithelial progenitor/stem cells. Stem Cells.

[23] Tseng S.C. (2016). HC-HA/PTX3 purified from amniotic membrane as novel regenerative matrix: Insight into relationship between inflammation and regeneration. Investig. Ophthalmol. Vis. Sci..

[24] Dua H.S., Gomes J.A., King A.J., Maharajan V.S. (2004). The amniotic membrane in ophthalmology. Surv. Ophthalmol..

[25] Bouchard C.S., John T. (2004). Amniotic membrane transplantation in the management of severe ocular surface disease: Indications and outcomes. Ocul. Surf..

[26] He H., Tan Y., Duffort S., Perez V.L., Tseng S.C. (2014). In vivo downregulation of innate and adaptive immune responses in corneal allograft rejection by HC-HA/PTX3 complex purified from amniotic membrane. Investig. Ophthalmol. Vis. Sci..

[27] Zhu Y.T., Li F., Zhang Y., Chen S.Y., Tighe S., Lin S.Y., Tseng S.C.G. (2020). HC-HA/PTX3 purified from human amniotic membrane reverts human corneal fibroblasts and myofibroblasts to keratocytes by activating BMP signaling. Investig. Ophthalmol. Vis. Sci..

[28] Papanna R., Moise Jr K.J., Mann L.K., Fletcher S., Schniederjan R., Bhattacharjee M.B., Stewart R.J., Kaur S., Prabhu S.P., Tseng S.C.G. (2016). Cryopreserved human umbilical cord patch for in-utero spina bifida repair. Ultrasound Obstet. Gynecol. Off. J. Int. Soc. Ultrasound Obstet. Gynecol..

[29] Papanna R., Mann L.K., Snowise S., Morales Y., Prabhu S.P., Tseng S.C., Grill R., Fletcher S., Moise K.J., Papanna R. (2016). Neurological outcomes after human umbilical cord patch for in utero spina bifida repair in a sheep model. AJP Rep..

[30] Mann L.K., Won J.H., Trenton N.J., Garnett J., Snowise S., Fletcher S.A., Tseng S.C.G., Diehl M.R., Papanna R. (2019). Cryopreserved human umbilical cord versus acellular dermal matrix patches for in utero fetal spina bifida repair in a pregnant rat model. J. Neurosurg. Spine.

[31] Papanna R., Fletcher S., Moise K.J., Mann L.K., Tseng S.C. (2016). Cryopreserved human umbilical cord for in utero myeloschisis repair. Obstet. Gynecol..

[32] Joyeux L., Engels A.C., Van Der Merwe J., Aertsen M., Patel P.A., Deprez M., Khatoun A., Pranpanus S., Da Cunha M.G.M.C.M., De Vleeschauwer S. (2019). Validation of the fetal lamb model of spina bifida. Sci. Rep..

[33] Brown E.G., Saadai P., Pivetti C.D., Beattie M.S., Bresnahan J.C., Wang A., Farmer D.L. (2014). In utero repair of myelomeningocele with autologous amniotic membrane in the fetal lamb model. J. Pediatr. Surg..

[34] Levine G.J., Levine J.M., Budke C.M., Kerwin S.C., Au J., Vinayak A., Hettlich B., Slater M.R. (2009). Description and repeatability of a newly developed spinal cord injury scale for dogs. Prev. Vet. Med..

[35] Olby N.J., De Risio L., Munana K.R., Wosar M.A., Skeen T.M., Sharp N.J., Keene B.W. (2001). Development of a functional scoring system in dogs with acute spinal cord injuries. Am. J. Vet. Res..

[36] Stokes B.T., Noyes D.H., Behrmann D.L. (1992). An electromechanical spinal injury technique with dynamic sensitivity. J. Neurotrauma.

[37] Levine J.M., Levine G.J., Kerwin S.C., Hettlich B.F., Fosgate G.T. (2006). Association between various physical factors and acute thoracolumbar intervertebral disk extrusion or protrusion in Dachshunds. J. Am. Vet. Med Assoc..

[38] Mangena M., Gcebe N., Pierneef R., Thompson P.N., Adesiyun A.A. (2021). Q Fever: Seroprevalence, risk factors in slaughter livestock and genotypes of coxiella burnetii in South Africa. Pathogens.

[39] Miller K.L., Stagg C.J., Douaud G., Jbabdi S., Smith S.M., Behrens T.E., Jenkinson M., Chance S.A., Esiri M.M., Voets N.L. (2011). Diffusion imaging of whole, post-mortem human brains on a clinical MRI scanner. Neuroimage.

[40] Alam H., Sehgal L., Kundu S.T., Dalal S.N., Vaidya M.M. (2011). Novel function of keratins 5 and 14 in proliferation and differentiation of stratified epithelial cells. Mol. Biol. Cell..

[41] Caplan L.R., Norohna A.B., Amico L.L. (1990). Syringomyelia and arachnoiditis. J. Neurol. Neurosurg. Psychiatry.

[42] Park Y.K., Tator C.H. (1998). Prevention of arachnoiditis and postoperative tethering of the spinal cord with Gore-Tex surgical membrane: An experimental study with rats. Neurosurgery.

[43] Darby I.A., Zakuan N., Billet F., Desmouliere A. (2016). The myofibroblast, a key cell in normal and pathological tissue repair. Cell. Mol. Life Sci..

[44] Liddelow S.A., Barres B.A. (2017). Reactive astrocytes: Production, function, and therapeutic potential. Immunity.

[45] Caldarelli M., Boscarelli A., Massimi L. (2013). Recurrent tethered cord: Radiological investigation and management. Child’s Nerv. Syst. ChNS Off. J. Int. Soc. Pediatric Neurosurg..

[46] Heiss J.D., Snyder K., Peterson M.M., Patronas N.J., Butman J.A., Smith R.K., DeVroom H.L., Sansur C.A., Eskioglu E., Kammerer W.A. (2012). Pathophysiology of primary spinal syringomyelia. J. Neurosurg. Spine.

[47] Heiss J.D., Patronas N., DeVroom H.L., Shawker T., Ennis R., Kammerer W., Eidsath A., Talbot T., Morris J., Eskioglu E. (1999). Elucidating the pathophysiology of syringomyelia. J. Neurosurg..

[48] Oldfield E.H., Muraszko K., Shawker T.H., Patronas N.J. (1994). Pathophysiology of syringomyelia associated with Chiari I malformation of the cerebellar tonsils. Implications for diagnosis and treatment. J. Neurosurg..

[49] Galganski L.A., Kumar P., Vanover M.A., Pivetti C.D., Anderson J.E., Lankford L., Paxton Z.J., Chung K., Lee C., Hegazi M.S. (2020). In utero treatment of myelomeningocele with placental mesenchymal stromal cells—Selection of an optimal cell line in preparation for clinical trials. J. Pediatric Surg..

[50] Stavrinou P., Kunz M., Lehner M., Heger A., Muller-Felber W., Tonn J.-C., Peraud A. (2011). Children with tethered cord syndrome of different etiology benefit from microsurgery-a single institution experience. Child’s Nerv. Syst. ChNS Off. J. Int. Soc. Pediatric Neurosurg..

[51] Wang A., Brown E.G., Lankford L., Keller B.A., Pivetti C.D., Sitkin N.A., Beattie M.S., Bresnahan J.C., Farmer D. (2015). Placental mesenchymal stromal cells rescue ambulation in ovine myelomeningocele. Stem Cells Transl. Med..

[52] Sanchez e Oliveira Rde C., Valente P.R., Abou-Jamra R.C., Araujo A., Saldiva P.H., Pedreira D.A. (2007). Biosynthetic cellulose induces the formation of a neoduramater following pre-natal correction of meningomyelocele in fetal sheep. Acta Cir. Bras..

[53] Sayad W.Y., Harvey S.C. (1923). The Regeneration of the meninges: The dura mater. Ann. Surg..

[54] Lear M., Harvey S.C. (1924). The Regeneration of the meninges. Ann. Surg..

