# Supplementary material for: Allografts for Skin Closure during In Utero Spina Bifida Repair in a Sheep Model
Source: J Clin Med. 2021 Oct 25;10(21):4928. doi: 10.3390/jcm10214928 (PMC8584988; doi:10.3390/jcm10214928)
Supplement: Supplementary file 1 [file jcm-10-04928-s001.zip › jcm-1404736-supplementary.pdf]

Supplemental Materials:

Supplemental Figures

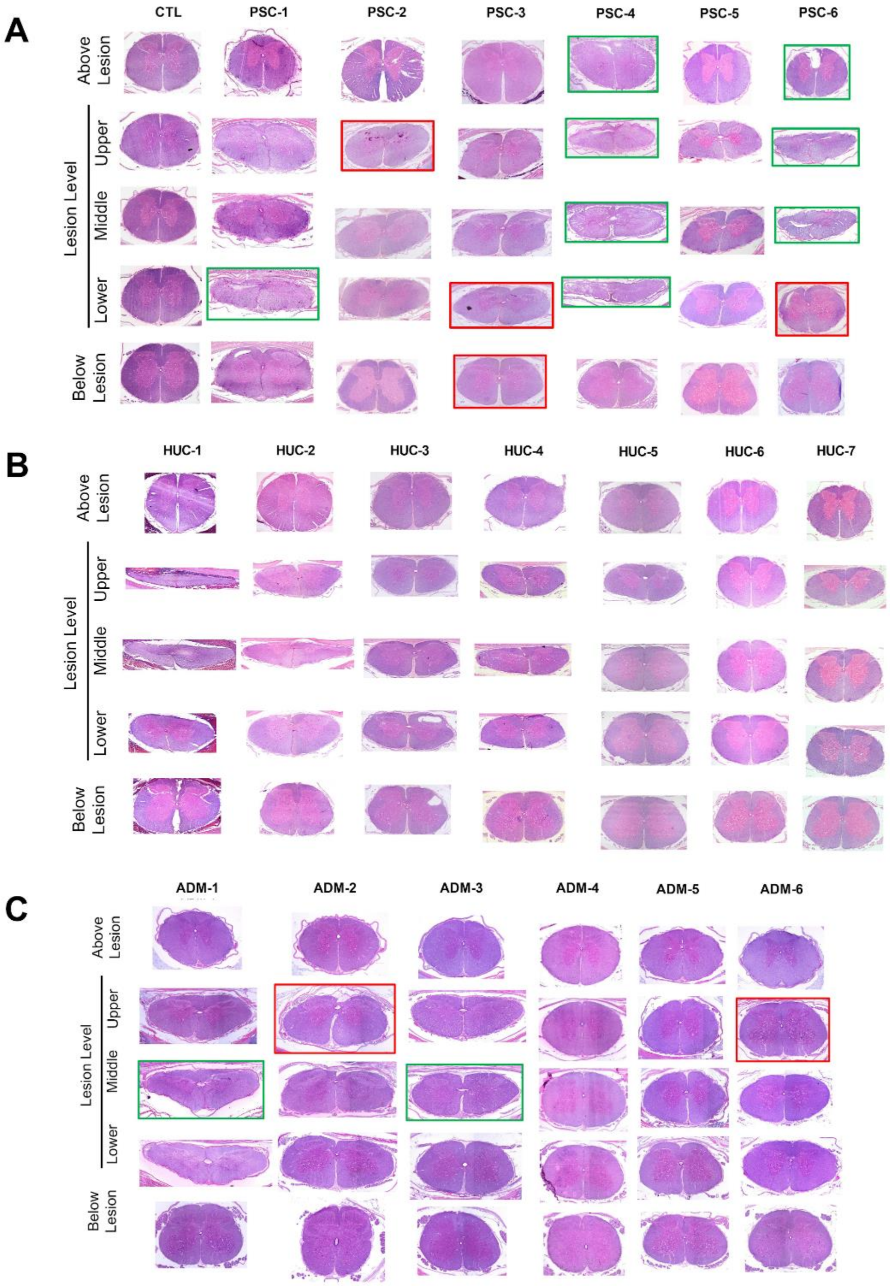

Figure S1. Five level histology of the spine to assess for syringomyelia and hemorrhage. H&E sections were evaluated at five levels for all spines, this those being coded as 'above lesion', 'upper lesion', 'middle lesion', 'lower lesion' and 'below lesion'. The syringomyelia present within spinal cord sections are enclosed with a green border, and if a hemorrhagic event occurred by red border. (**A**) Control and PSC lambs' spinal sections are presented. (**B**) HUC lambs' H&E sections are provided, including the HUC-1 lamb that had an acute hemorrhage at the time of delivery leading to poor/outlier scores in the HUC group. (**C**) Histological data for the ADM repair are depicted here.

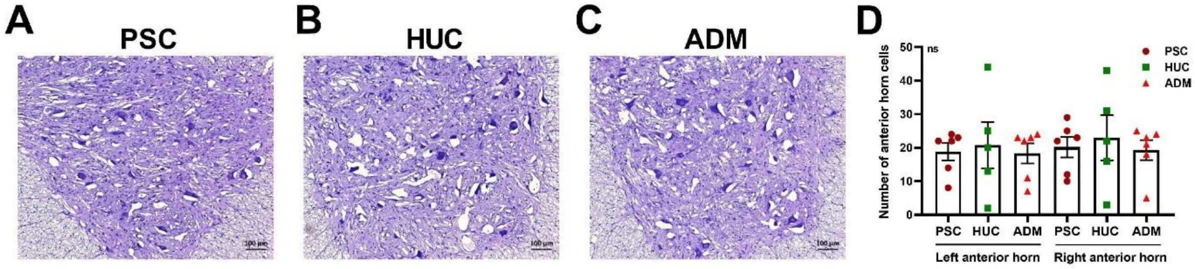

Figure S2. Anterior horn cell numbers are equally distributed amongst all defect repair sites of different repair methodologies. Anterior horn cells present in the middle of defect repair site of lambs' spinal cords repaired (**A**) conventionally (PSC), or with a (**B**) HUC patch, or with a (**C**) ADM patch. (**D**) Anterior horn cells counts are graphically represented on the left anterior horn and on the right anterior horn per repair performed. Significance was determined by ANOVA, Kruskal-Wallis test, Two-stage linear step-up procedure of Benjamini, Krieger and Yekutieli.

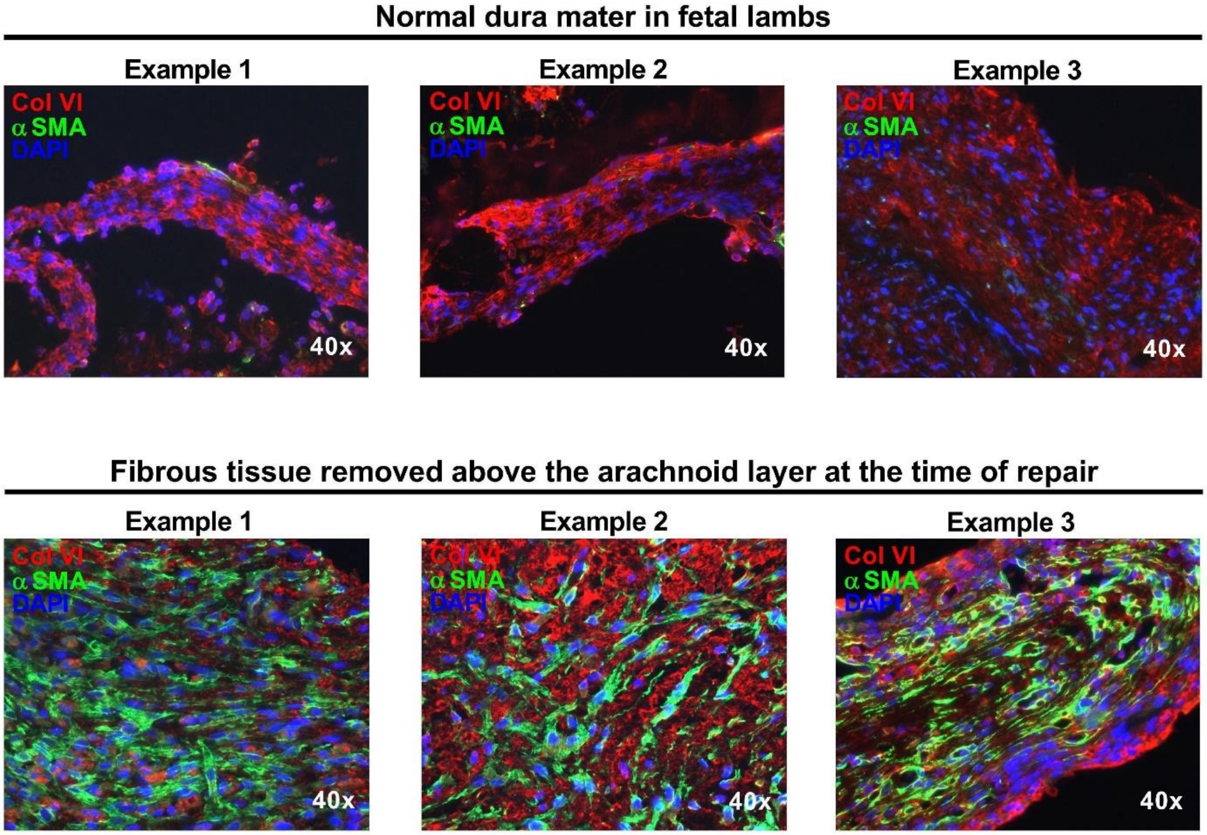

Figure S3. Fibrous tissue removed at defect repair sites prior to repair during surgery is scar tissue. The upper panel depicts the dura of three different lambs without SB defects, whereas the lower panel illustrates fibrous tissue akin to scar formation at defect repair sites prior to surgical repair in three lamb fetuses. All sections were stained immunofluorescently with anti-Col VI, anti-αSMA, and DAPI.

Supplemental Tables

Table S1. MRI Settings for the acquisition of lambs' spinal cords at defect repair sites.

Weighting	Acquisition Dimension	Protocol	Repetition Time (ms)	Echo Time (ms)	RARE Factor	Average	Flip Angle Degree
**T1**	3D	Rapid Acquisition with Relaxed Enhancement (RARE)	500	24	4	4	90
**T2**	3D	RARE	2000	60	8	3	90
**T2***	2D	Fast Low Angle Shot	4200	10	-	10	18
